# Endless Journey of Adenosine Signaling in Cardioprotective Mechanism of Conditioning Techniques: Clinical Evidence

**DOI:** 10.2174/1573403X19666230612112259

**Published:** 2023-10-02

**Authors:** Kuldeep Kumar, Nirmal Singh, Harlokesh Narayan Yadav, Leonid Maslov, Amteshwar Singh Jaggi

**Affiliations:** 1 Department of Pharmaceutical Sciences and Drug Research, Punjabi University, Patiala, Punjab 147002, India;; 2 Department of Pharmacology, All India Institute of Medical Sciences (AIIMS), New Delhi 110029, India;; 3 Cardiology Research Institute, Tomsk National Research Medical Center of the Russian Academy of Science, Tomsk, Russia

**Keywords:** Adenosine, cardiovascular disorders, ischemia-reperfusion injury, myocardial ischemia, preconditioning, postconditioning

## Abstract

Myocardial ischemic injury is a primary cause of death among various cardiovascular disorders. The condition occurs due to an interrupted supply of blood and vital nutrients (necessary for normal cellular activities and viability) to the myocardium, eventually leading to damage. Restoration of blood supply to ischemic tissue is noted to cause even more lethal reperfusion injury. Various strategies, including some conditioning techniques, like preconditioning and postconditioning, have been developed to check the detrimental effects of reperfusion injury. Many endogenous substances have been proposed to act as initiators, mediators, and end effectors of these conditioning techniques. Substances, like adenosine, bradykinin, acetylcholine, angiotensin, norepinephrine, opioids, *etc*., have been reported to mediate cardioprotective activity. Among these agents, adenosine has been widely studied and suggested to have the most pronounced cardioprotective effects. The current review article highlights the role of adenosine signaling in the cardioprotective mechanism of conditioning techniques. The article also provides an insight into various clinical studies that substantiate the applicability of adenosine as a cardioprotective agent in myocardial reperfusion injury.

## 1 INTRODUCTION

Cardiovascular disorders (CVDs), such as myocardial infarction, heart failure, stroke, and coronary artery disease, are the primary cause of mortality worldwide. A report published by World Health Organization (WHO) in 2019 states that around 17.9 million people die yearly due to CVD, representing approximately 32% of global mortality. It has also been reported that this mortality rate can rise to 23.3 million by 2030 [1]. In addition, about 12.7% of global mortality is due to ischemic heart disease (IHD) in developing countries [2, 3].

Ischemia is a condition of restricted blood supply to the tissue that eventually leads to the shortage of oxygen and other nutrients vital for normal cellular metabolism and viability [4, 5]. Ischemia can be categorized as myocardial ischemia, cerebral ischemia, renal ischemia, lung ischemia, hepatic ischemia, intestinal ischemia, *etc.* [6]. Restoration of blood flow to the ischemic organ is perhaps the only way to prevent the ischemic organ from irreversible tissue damage. Paradoxically, rapid resumption of blood flow to the infarcted organ itself induces the death of cells of the organ and exacerbates the extent of injury of that particular organ, referred to as ischemia-reperfusion (IR) injury [7]. The concept of IR injury was first introduced in 1960, and is nowadays encountered in many surgical as well as non-surgical manifestations, such as organ transplantation, cardiopulmonary bypass surgery, aneurysm repair, stroke, myocardial infarction, trauma, shock, hemorrhage [8], traumatic head injury, carotid endarterectomy, and deep hypothermic circulatory arrest [9].

Several therapeutic strategies, like primary percutaneous coronary intervention (PPCI) and thrombolytic approaches involving the new generation of anti-platelet drugs and anti-thrombotic agents, are applied clinically to manage ischemic injury [10, 11]. However, until today, neither pharmacological nor non-pharmacological interventions have successfully protected the organ against IR injury [12].

Over the last decade, some conditioning techniques involving brief episodes of ischemia followed by reperfusion either before (preconditioning) or after (postconditioning) the prolonged ischemia in the affected region have been developed to mitigate IR injury [13-15]. Remote preconditioning and remote postconditioning are modified conditioning methods where brief episodes of ischemia-reperfusion are applied at distant organs to protect the ischemic organ [16, 17].

Pharmacological conditioning (preconditioning or postconditioning) is another practical approach to contain IR injury. In pharmacological conditioning, a therapeutic agent is administered before or after the significant ischemic event. Although this therapeutic agent gets eliminated from the body, it eventually activates several initiators, mediators, and triggers that confer protection to the organ from IR injury [18]. Several pharmacological agents, such as adenosine [19], bradykinin [20], acetylcholine [20, 21], angiotensin-II [22], opioids [23], norepinephrine [24], platelet-activating factor (PAF) [25], nicotinamide [26], phenylephrine [27], endothelin [28], leptin [29], insulin-growth factor-I (IGF-I) [30], natriuretic peptides [31], urocortin [32], fibroblast growth factor-I and II (FGF-I and II) [16], *etc.* have been reported to provide cardioprotective effects in myocardial IR injury when administered exogenously. Some studies reveal that pharmacological conditioning is a safer way of protecting against IR injury [33-35].

## 2 OVERVIEW OF THE PATHOPHYSIOLOGY OF IR INJURY

Myocardial ischemia is encountered in many pathological situations, as discussed above. Longer duration ischemia contributes to a variety of cellular, metabolic, and ultra-structural changes, such as altered membrane potential, increased intracellular calcium and sodium overload [36], cellular swelling, increased hypoxanthine, and decreased adenosine triphosphate (ATP), phosphocreatine and glutathione levels. During the ischemic phase, aerobic metabolism of the heart switches to anaerobic metabolism, followed by decreased oxidative phosphorylation that subsequently causes failure in the re-synthesis of energy-rich molecules, such as ATP and phosphocreatine. Moreover, it also increases the accumulation of hypoxanthine in the heart. In addition, ATP-operated ion channels’ function is also impaired that further leads to the increased entry of calcium, sodium, and water into the cell [37, 38]. During normal physiology, hypoxanthine gets oxidized to xanthine by xanthine dehydrogenase, but during the ischemic phase, xanthine dehydrogenase gets converted into xanthine oxidase. Xanthine dehydrogenase utilizes nicotinamide adenine dinucleotide (NAD) as its substrate, while xanthine oxidase uses oxygen as its substrate. Therefore, during the ischemic phase, xanthine oxidase is unable to convert the hypoxanthine to xanthine, subsequently causing excess accumulation of hypoxanthine in the heart. In addition to this, during the reperfusion phase, oxygen re-enters the heart, which causes the conversion of excess hypoxanthine (accumulated during the ischemic phase) to xanthine by xanthine oxidase [39]. This process further results in the generation of large amounts of reactive oxygen species (ROS), such as superoxide anion (O^2-^), hydrogen peroxide (H_2_O_2_), and hydroxyl radical (OH^-^) [40]. This free radical activates the intracellular signalling pathway and causes membrane injury, finally leading to myocardial IR injury [41]. In addition to this, during reperfusion, oxygen is re-introduced into the cells that starts damaging cellular proteins as well as deoxyribonucleic acid (DNA), which are also other main factors of myocardial IR injury [42]. Some studies have shown oxidative stress, neutrophils, leukocyte activation, and excessive intracellular osmotic load together to be involved in the pathogenesis of myocardial IR injury [43, 44]. Besides these pathological mechanisms, several endogenous mediators, such as caspase-3, caspase-8 [45], calpains [46], interleukin-6 (IL-6) [47], and tumor necrosis factor-α (TNF-α) [48] also play an important role in the pathogenesis of myocardial IR injury.

## 3 VARIOUS ENDOGENOUS AND EXOGENOUS CARDIOPROTECTIVE MOLECULES

### 3.1 Physical stimuli

Myocardial stretch results in the activation of gadolinium-sensitive sarcolemmal channels as well as certain downstream signaling (adenosine, PKC) and K_ATP_ channels, and eventually leads to a reduction in the infarct size [49]. Additionally, mild hypothermia during the ischemic phase is speculated to exhibit protection against infarction through various mechanisms, including slowing energy metabolism and activation of different protective signaling pathways, notably ERK1/2 [50]. On the contrary, hyperthermia has been documented to provide delayed protection through the up-regulation of heat shock proteins [51].

### 3.2 Chemical stimuli

Several endogenous chemical stimuli, such as calcium ions, ROS, reactive nitrogen species (RNS), hydrogen sulfide, and more classical ligands that activate sarcolemmal receptors, have been found to elicit cardioprotection. Among these, the extracellular calcium ions provide preconditioning effects *via* adenosine and PKC activation [52, 53]. ROS acts as a double-edged sword in the conditioning phenomena; the excess formation of ROS leads to irreversible injury, whereas small amounts of ROS generated in response to mitochondrial K_ATP_ channel activation or mitochondrial permeability transition pore opening contribute towards protection [54]. RNS, notably NO, also work in the same dose-dependent manner [55]. In small concentrations, they improve ventricular contractile function [56] as well as oxygen consumption [57]. On the contrary, high concentrations of NO disrupt cardiac functioning [58]. In addition to this, exogenous NO can elicit a preconditioning response, but endogenous NO does not confer the same in its acute form [59]. Moreover, endogenous NO also plays an intricate role in the protective phenomenon of postconditioning [60] and remote preconditioning [61]. Besides NO, some other gaseous molecules, such as hydrogen sulfide and carbon monoxide, have also been reported to possess cardioprotective potential by reducing infarct size [62].

### 3.3 Autacoids

It has been documented that during the preconditioning IR cycle(s), cardiomyocytes, endothelium, and interstitial cells release some autacoids, including adenosine and bradykinin [63]. Adenosine binds to its various receptors (such as A1, A_2A_, A_2B_, and A3) and exhibits cardioprotection by activating/deactivating different downstream signaling pathways [64]. Bradykinin is cleaved from kininogen precursors in the interstitium and catabolized through the angiotensin-converting enzyme in the vasculature, but also neutral endopeptidase in the interstitium [65, 66]. It is speculated that there is a rapid increase in the bradykinin levels during the preconditioning IR cycle(s) [63] that further activates the bradykinin receptor 2 subtype on cardiomyocytes, and eventually causes the activation of the downstream endothelial NOS/PKG and RISK pathways [67]. Bradykinin also decreases infarct size by increasing cyclo-oxygenase (COX) and prostacyclin synthesis [68, 69].

### 3.4 Neurohormones

Exogenous administration of certain neurotransmitters and hormones, such as acetylcholine [20, 70], angiotensin [71], catecholamines, endothelin [72, 73], and opioids, can induce cardioprotection through activation of their respective receptors. Catecholamines act through α- [74, 75] and β-adrenoceptor [76] activation. However, only α-adrenoceptor activation is mainly found to be involved in the conditioning phenomenon *via* the formation of adenosine [75, 77]. Apart from their acute release from nerve endings, opioids are also synthesized in cardiomyocytes. In adult cardiomyocytes, opioids activate δ and k receptors, which couple to Gi proteins, and thus mutually share the downstream signaling with adenosine and bradykinin [78, 79].

### 3.5 Peptide Hormones

Several peptide hormones, such as adrenomedullin [80], natriuretic peptides [81, 82], urocortins [83], and leptin [84] have been documented to confer protection against myocardial IR injury on exogenous administration. Additionally, these peptide hormones have been reported to act in the postconditioning through various signal transduction elements, such as NO [85], cGMP, mitochondrial K_ATP_ channels [81, 82], and RISK [83]. Moreover, exogenous glucagon-like peptides 1 and 2 act by activating their Gs-coupled receptor and subsequently triggering PKA and the RISK pathway to reduce infarct size [86, 87], but their involvement has not been established in endogenous conditioning.

### 3.6 Growth Factors

Growth factors, such as insulin-like growth factor-1 [88], fibroblast growth factor-1 [89], and fibroblast growth factor-2 [90] cause a reduction in infarct size after exogenous administration, but no evidence supports the involvement of growth factors in ischemic preconditioning, ischemic postconditioning, and remote ischemic conditioning.

### 3.7 Cytokines/Chemokines

Besides peptide hormones and growth factors, cytokines/chemokines also have been found to play a crucial role in ischemic preconditioning and postconditioning. On exogenous administration, the prototypic cytokine, *i.e.*, TNF-α, not only reduces the infarct size [91], but is also essential for ischemic pre and postconditioning [92, 93]. Activation of TNF receptor-2, STAT-3, and mitochondrial K_ATP_-channels is the major element that mediates the protection [91, 94].

## 4 ADENOSINE

Adenosine is a purine nucleoside composed of an adenine molecule attached to a ribose sugar moiety *via* a beta-N9-glycosidic bond [95]. In the intracellular space, adenosine is synthesized through purine synthesis, and extracellularly, it is accumulated as a metabolite of adenosine triphosphate (ATP) [96]. Intracellular concentrations of adenosine have been speculated to rise in situations of an interruption between ATP synthesis and its use (*e.g.*, ischemia, cellular stress, IR injury, inflammation, *etc.*) [97, 98]. It is well-documented that adenosine modulates several physiological functions, such as blood flow, heart rate, contractility, and vasodilatation [99, 100]. Additionally, exogenous administration of adenosine triggers the activation of several beneficial metabolic processes that confer protection in the settings of IR injury [101]. Moreover, this exogenously administered adenosine also contributes to the replenishment of diminished levels of ATP in viable ischemic cells [102]. Besides, several reports have documented that adenosine plays an intricate role in the repairing of damaged blood vessels as well as in the process of angiogenesis [103, 104] by increasing the release of angiogenic mediators, including IL-8, fibroblast growth factor (FGF), vascular endothelial growth factor (VEGF), *etc.* [105].

### 4.1 Role of Adenosine in Cardioprotection

Adenosine plays a vital role in the regulation of vascular tone in the arterial tree by causing the relaxation of arterial smooth muscle [106, 107] that further decreases vascular resistance and eventually facilitates the flow of blood and oxygen [108]. The actions of adenosine on coronary blood flow are speculated to be mediated primarily through the activation of A2A receptors [109]. Additionally, adenosine binds to and activates A2A receptors, which trigger the opening of Kv and K_ATP_ channels present in smooth muscle cells, resulting in membrane hyperpolarization and relaxation [110, 111]. Moreover, adenosine causes the release of NO from endothelium that mediates the dilatation of coronary arteries [112]. Besides, adenosine can be used as a powerful tool in myocardial perfusion imaging studies for pharmacological stress testing [113]. It is also well established that endothelial cells line the luminal surface of the blood vessels, which is found to be involved in the regulation of blood flow, exchange of nutrients, passage of waste products, and control of thrombosis/thrombolysis [114]. The endothelial cells in the vasculature produce NO (a potent vasodilator) in a reaction catalyzed by NO synthase. A2A receptors’ stimulation triggers the endothelial nitric oxide synthase (eNOS) activation that eventually leads to increased synthesis of NO in human endothelium [115].

### 4.2 Adenosine Receptors and their Signaling

Adenosine exerts effects by binding to four different types of G-protein-coupled receptors, *i.e.,* A_1_R, A_2A_R, A_2B_R, and A_3_R [116, 117]. On a wide variety of cells, these receptors modulate intracellular levels of cAMP by stimulating or inhibiting adenyl cyclase (AC) activity. A_1_ and A_3_ receptors signal by inhibiting AC activity that further down-regulates cAMP. On the other side, A_2A_ and A_2B_ receptors signal by stimulating AC activity that further up-regulates cAMP [95, 118, 119], subsequently causing potent vasodilatation of the coronary blood vessels and eventually increasing blood flow to the myocardium [120, 121]. In addition, increased activation of the adenylate cyclase pathway also results in decreased superoxide generation and neutrophil activation, aggregation, and adherence to the endothelium [121, 122]. The mechanisms mentioned above further reduce the neutrophilic arterial plugging phenomenon, a critical pathological pathway of reperfusion injury. Decreased activation and aggregation of neutrophils further inhibit inflammatory reactions (a significant step of reperfusion injury) by preventing the release of pro-inflammatory mediators [121]. In addition to these classical pathways, adenosine receptor signaling can also activate other pathways, including phospholipase-C (PLC) [123], phosphatidylinositol-3-kinase (PI3K) [124], mitogen-activated protein kinase (MAPK’s) [124, 125], extracellular signal-regulated kinase 1/2 (ERK1/2) [126, 127], and Akt [127] pathways, to influence gene expression [128]. Several studies have revealed that adenosine shows its highest affinity for A_1_ and A_2A_ receptors, an intermediate affinity for the A_3_ receptor, and the lowest affinity for the A_2B_ receptor [129, 130].

## 5 CARDIOPROTECTIVE SIGNALING PATHWAYS TRIGGERED BY ADENOSINE

Studies have reported all adenosine receptors, *i.e.*, A_1_, A_2A_, A_2B_, and A_3_, to be distributed in heart and blood vessels. It has also been documented that A_1_ and A_2A_ receptors are expressed in adult ventricular myocytes, but the presence of A_2B_ and A_3_ receptors at the same site does not support the evidence [131, 132]. Therefore, the protective actions of A_2B_ and A_3_ receptors on myocardial cells against ischemia do not relate to the direct myocyte response. It may be due to action on other types of cells. The A_1_ receptor is the most extensively studied adenosine receptor subtype in the context of myocardial protection. Moreover, it has been reported that the A_3_ receptor triggers cardioprotective action like the A_1_ receptor, but *via* different signaling mechanisms. In addition, A_2A_ and A_2B_ receptors also have gained immense popularity for their cardioprotective roles in various myocardial pathologies [133, 134].

### 5.1 Cardioprotective Signaling Pathways Triggered by Adenosine A_1_ Receptor (A_1_AR)

A_1_AR is suggested to play a pivotal role in myocardial protection. A_3_AR, being discovered later than A1 and A2AR subtypes, is ignored in terms of myocardial protection, except in some work done by a few researchers [135, 136]. Studies have shown that A_1_AR exerts its cardioprotective effects by GPCR-coupled protein kinase (PK) signaling mechanism [137]. Despite this, some controversies exist regarding cardioprotective signaling mechanisms and cardioprotective responses mediated by A_1_AR. Earlier, it was hypothesized that cardioprotective effects of A_1_AR are due to decreased adenosine triphosphate (ATP) depletion or increased nucleotide repletion on reperfusion, stimulation of glycolysis, and normalization of oxygen demand/supply ratio [138, 139]. Subsequent studies have reported the vital role of PK signaling [140, 141] along with numerous secondary end-effectors, *i.e.,* mitochondrial K_ATP_ channel [142, 143], mitochondrial permeability transition pore (mPTP), *etc.* [144], in myocardial protection mediated *via* A_1_AR preconditioning.

**Fig. (1):**
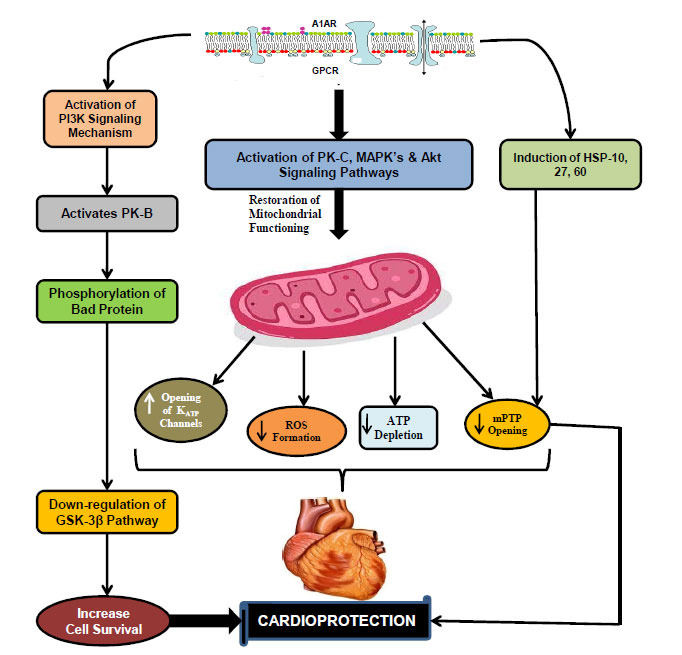
Cardioprotective signaling mechanisms triggered by adenosine A_1_ receptor. **Abbreviations:** A1AR: A1 adenosine receptor; ATP: Adenosine triphosphate; GPCR: G-protein coupled receptor; GSK-3β: Glycogen synthase kinase; HSP: Heat shock protein; K_ATP_: ATP-sensitive potassium channel; MAPK: Mitogen activated protein kinase; mPTP: Mitochondrial permeability transition pore; PI3K: Phosphoionositol-3-kinase; PK-C: Protein kinase-C; PK-B: Protein kinase-B; ROS: Reactive oxygen species.

Conventional pre-clinical studies have established a connection between A_1_AR agonism and many kinase systems, such as PKC [123], MAPKs [123, 141], PI3K, and Akt [127]. It has already been proven that the aforementioned signaling pathways may be involved individually or may work together in connection with secondary effectors mechanisms, *i.e.,* mPTP, K_ATP_ channels, *etc.,* for showing their cardioprotective responses [142-144]. However, specific kinase involvement in cardioprotection still remains contentious. Several studies support the involvement of PKC in A_1_AR-mediated cardioprotection by using some PKC inhibitors [144-146], but the actual effects of A_1_AR on PKC expression and translocation are poorly documented [147]. Schulte and colleagues conducted a study to clarify confusion regarding kinase involvement in cardioprotection. This study found that non-specific kinase signaling inhibitors, including PKC inhibitors, could significantly abolish the effects of A_1_AR agonists [125]. In addition, it is also reported that A_1_AR activates other cardioprotective pathways, such as PI3K [148], through the transactivation of tyrosine kinase [149]. It further activates downstream targets *i.e.*, Akt/PKB [127, 148], which play a pivotal role in cell survival through different mechanisms, such as phosphorylation of Bad protein and down-regulation of proapoptotic glycogen synthase kinase-3β (GSK-3β) pathway [127, 141, 150]. Besides this, several heat shock proteins (HSP), such as HSP-10, HSP-27, and HSP-60, are also induced by these kinases that further target mitochondrial signaling, which eventually provides cardioprotection by limiting mPTP [151, 152]. Moreover, a few studies have also demonstrated the role of MAPK in A_1_AR-mediated cardioprotection by utilizing MAPK signaling inhibitors [153-155] (Fig. **1**).

### 5.2 Cardioprotective Signaling Pathways Triggered by Adenosine A_2A_ and Adenosine A_2B_ Receptors

Adenosine A_2_ receptor subtypes are not explored much in the context of myocardial IR injury. Despite this, it has been suggested that the A2 receptor plays a vital role in the modulation of cardiovascular stress and inflammatory processes. It has been documented that both A_2_ sub-types receptors exhibit immune-modulatory and anti-inflammatory responses that eventually act as a protective mechanism in case of ischemia and IR injury [134, 156, 157]. A study by Jorden and co-workers was the first to report the cardioprotective actions of A_2A_R. They revealed that A_2_AR mediates cardioprotection *via* inhibition of neutrophil activation and neutrophil-vascular interactions [43, 157].

#### 5.2.1 A_2A_AR-mediated Cardioprotection

**Fig. (2):**
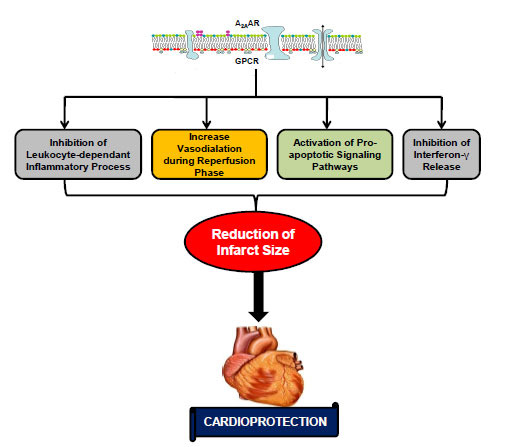
Cardioprotective signaling mechanisms activated by adenosine A_2A_ receptor. **Abbreviations:** A_2A_AR: A_2A_ adenosine receptor; GPCR: G-protein coupled receptor.

It has been documented that A_2A_AR plays an intricate role in regulating several inflammatory and immune responses in various organs [155, 157, 158]. Inflammation is a crucial pathological hallmark involved in both the early and late phases of IR injury and remodeling associated with IR injury. Based on the above fact, it is assumed that A_2A_AR exhibits protective action in IR injury of various organs. The signaling pathways that are associated with cardioprotective activities of A_2A_AR include inhibition of leukocyte-dependent inflammatory processes [159, 160] and a direct inotropic action [161] that appear selective for post-ischemic tissue [162]. In addition, it is reported by various animal experimental models that cardioprotection mediated *via* A_2A_AR may be due to increased vasodilatation during the reperfusion phase [140, 163,]. Furthermore, few *in vivo* studies have also reported the reduction of IR injury mediated *via* A_2A_AR-dependent activation of neutrophil [157, 158, 160] and pro-apoptotic [164, 165] signaling pathways. Another study documented the inhibition of the release of interferon-γ (INF-γ) mediated *via* A_2A_AR agonism that further reduced the infarct size [166] (Fig. **2**).

#### 5.2.2 A_2B_AR-triggered Cardioprotection

**Fig. (3):**
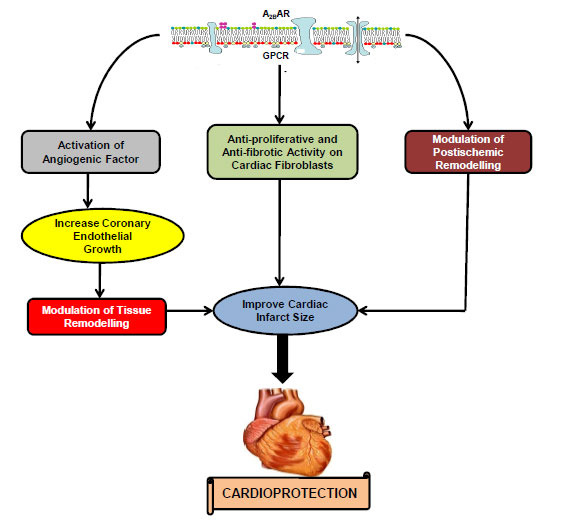
Cardioprotective signaling mechanisms mediated by A_2B_ adenosine receptor. **Abbreviations:** A_2B_AR: A_2B_ adenosine receptor; GPCR: G-protein coupled receptor.

The A_2B_AR is poorly understood and less explored than A_2A_AR in the context of myocardial IR injury. Moreover, no study has reported the direct expression of A_2B_AR on cardiomyocytes, unlike A_2A_AR [131, 132, 167]. Hence, the cardioprotective effects of A_2B_AR may be due to other types of cell involvement. Studies suggest that the A_2B_AR activates angiogenic factors [168-170], and further causes coronary endothelial growth [103, 169, 170]. Moreover, A_2B_AR also plays a vital role in the modulation of vascular growth and tissue remodeling [168, 171]. Few studies have reported the anti-proliferative and anti-fibrotic functions of A_2B_AR on cardiac fibroblasts and other organ cells [171, 172]. Some more studies have also documented the role of A_2B_AR in the modulation of post-ischemic remodeling and reported its role in the infarct-sparing effects of postconditioning [173, 174] (Fig. **3**).

### 5.3 A_3_AR-triggered Cardioprotection

**Fig. (4):**
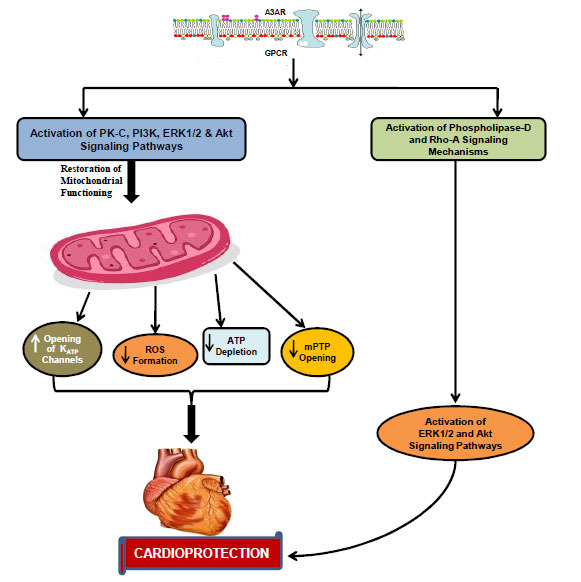
Signaling mechanisms triggered during A_3_ adenosine receptor-mediated cardioprotection. **Abbreviations:** A_3_AR: A_3_ adenosine receptor; ATP: Adenosine triphosphate; ERK1/2: Extracellular receptor kinase; GPCR: G-protein coupled receptor; K_ATP_: ATP-sensitive potassium channel; mPTP: Mitochondrial permeability transition pore; PI3K: Phosphoionositol-3-kinase; PK-C: Protein kinase-C; ROS: Reactive oxygen species.

Since its discovery, A_3_AR has been shown to mediate similar cardioprotective effects in various species and models [175, 176]. It is documented that A_3_AR is similar to A_1_AR in exhibiting cardioprotective actions. The A_3_AR also produces its cardioprotective effects *via* activation of PKC [177, 178] PI3K [76, 125], ERK [76], and mitochondrial K_ATP_ channels [179, 180]. On the other side, a study on avian myocyte cells (which may or may not be similar to mammalian myocytes) revealed that A_3_AR shows its protective effects by triggering novel phospholipase-D and Rho-A pathways [181, 182]. Moreover, it is also documented that A_3_AR-mediated pathways, *i.e.,* phospholipase-D/Rho-A, produce less pronounced but more sustained activation of downstream kinases than the A_1_AR-coupled phospholipase-C pathway [181]. Other studies suggest that A_3_AR also causes the activation of ERK1/2 and Akt pathways in mammalian cardiomyocytes to produce protective effects [141, 150]. In addition, A3AR triggers acute protection and participates in the sustained effects of preconditioning [142] (Fig. **4**).

## 6 CLINICAL EVIDENCE OF ADENOSINE CONDITIONING AS A CARDIOPROTECTIVE STRATEGY IN MYOCARDIAL IR INJURY

### 6.1 Evidence

Several clinical trials have witnessed adenosine's usefulness as a pharmacological conditioning agent in IR injury. A single-center, randomized, placebo-controlled trial was conducted on 70 patients suffering from acute myocardial infarction (AMI) to evaluate the role of adenosine administration in immediate electrocardiography and angiography results during primary angioplasty. For this, patients were divided into two groups; one group received intracoronary adenosine through a catheter immediately after crossing the lesions of the infracted artery with wire and after the balloon's first inflation. Another group was treated as a placebo control group. After adenosine treatment, there was an improvement in the resolution of ST-segment elevation of adenosine-treated patients compared to the placebo group patients. In addition, adenosine also improved the borderline TIMI 3 flow after the PCI procedure in adenosine-treated patients compared to placebo group patients. Moreover, results were also analyzed regarding significant improvement in myocardial blush grade 3 at the end of the PCI procedure in adenosine group patients compared to placebo group patients [183].

Another blinded, randomized, placebo-controlled study investigated the effect of high-dose intracoronary adenosine on ST-segment elevation after PCI. A total of 51 patients were selected undergoing PCI for AMI and administered a high dose of intracoronary adenosine. After successful PCI with intracoronary administration of adenosine for AMI, more than 70% of patients showed persistent ST-segment elevation compared to PCI without adenosine administration. In addition, beneficial effects of PCI with adenosine administration were also observed in terms of significant improvement in TIMI frame count, myocardial blush grade, and resistance index in adenosine-treated patients as compared to patients without adenosine [184].

Wang and colleagues conducted another study on patients suffering from acute ST-segment elevation myocardial infarction (STEMI) to check the effect of intravenous adenosine administration as an adjunct to PCI. They evaluated the effects of adenosine administration with PCI on myocardial perfusion and segmental contractile function, and divided the patients into two groups, *i.e.,* patients who received intravenous adenosine and saline within 12 hours of STEMI. The protective effects of adenosine administration with PCI were observed in terms of significant improvement in capillary blood volume and myocardial blood velocity in adenosine-treated patients compared to saline group patients. Moreover, there was a significant improvement in myocardial blood flow in adenosine-treated patients compared to control group patients. In addition, adenosine administration also improved myocardial wall strain, strain rate, segmental ejection fraction, and global contractile function compared to the control group patients [185].

One more randomized controlled trial substantiated the effect of adenosine pretreatment on myocardial IR injury. In this study, 82 children were selected to undergo cardio-pulmonary bypass surgery (CPBS) to correct congenital heart defects. Patients were divided into control and adenosine pretreatment groups. Treatment group patients received adenosine at the dose of 2.45 mg/kg by infusion 10 min before CPBS, while control group patients received normal saline. The protective effects of adenosine pretreatment were assessed in terms of significant hypotension in adenosine-treated patients as compared to control group patients. No significant effect on heart rate was observed of adenosine pretreatment. However, the protective effect of adenosine pretreatment was supported by decreased postoperative levels of troponin-I in the treatment group compared to control group patients. In addition, it was also observed that adenosine-treated patients required fewer postoperative ionotropic agents than control group children, indicating better cardiac functioning by adenosine pretreatment [186].

Mahaffey and colleagues designed a prospective and open-label trial, *i.e.,* Asymptomatic Myocardial Ischemia in STroke and Atherosclerotic Disease (AMISTAD-1) to investigate the effect of the administration of adenosine as an adjunct to thrombolysis therapy in patients suffering from AMI. For this, 236 patients suffering from AMI were selected and randomized into adenosine and placebo group patients. Adenosine was administered at the dose of 70 μg/kg/min over 3 h within 6 hours of the onset of infarction. The beneficial effects of adenosine administration as an adjunct to thrombolysis were assessed in terms of determining infarct size by performing single-photon emission computed tomography (SPECT) imaging. At the end of the study, a significant reduction in infarct size in adenosine-treated patients was observed than in placebo group patients. In addition, the myocardial salvage index (MSI) was also found to be improved in adenosine-treated patients compared to control group patients [187].

Marzilli and co-workers conducted another study to investigate the safety and efficacy of adenosine administration as an adjunct to percutaneous transluminal coronary angioplasty (PTCA) in AMI patients. A total of 54 patients were selected to undergo primary PTCA to repair AMI and assigned to the intracoronary adenosine treatment and control group. PTCA was successful in both treatments as well as in control group patients. The beneficial effects of adenosine treatment as an adjunct to the PTCA were observed in the no-reflow phenomenon, and improved ventricular function has been observed in adenosine-treated patients compared to control group patients. Moreover, the protective effect of adenosine treatment was further supported by decreased creatine kinase levels (CK-MB) in adenosine-treated patients than in control group patients. In addition, it was also observed that adverse cardiac events were less in adenosine treatment group patients and high in the control group patients [188].

One more study investigated the protective effects of adjunctive therapy of adenosine on patients undergoing PCI to repair myocardial reperfusion injury. For this, 279 patients were recruited and divided into the patients who received intracoronary adenosine infusion during PCI (79 patients) and those who received only PCI (200 patients). Adenosine was administered through intracoronary infusion for 20 minutes during PCI. The protective effects of adenosine administration as an adjunct to PCI were assessed in terms of reduction of myocardial reperfusion injury (RI) and a drop in infarct expansion after PCI in adenosine-treated patients compared to the control group patients. Moreover, it was also found that the rate of occurrence of major adverse cardiac events (death and myocardial infarction) was lower in adenosine-treated patients than in control group patients after one month of PCI [189].

Micari and colleagues conducted another study to check the effects of intravenous adenosine administration on micro-vascular reflow in patients undergoing primary coronary stenting (PCS) to correct AMI. A total of 30 patients were involved and randomized to adenosine-treated and vehicle-treated patient groups. Adenosine was administered intravenously at the dose of 50-70 gm/kg/min for 3 hours to the treatment group patients, and the vehicle was administered to the control group patients. The beneficial effects of adenosine administration along with PCS were determined by performing myocardial contrast echocardiography to detect risk areas before and after PCS. It was observed that the risk area was similar in both adenosine and control group patients. Still, infarct size was smaller in the adenosine-treated patients than control group patients. In addition, these protective effects were also supported by higher micro-vascular blood volume in the risk area even after four weeks in adenosine-treated patients compared to control group patients [190].

In another prospective, double-blind study, adenosine was administered in continuation with cold blood cardioplegia to investigate myocardial protection. A total of 80 patients undergoing aortic valve replacement were selected and divided into four groups. Adenosine was administered at the dose of 400 μmol/liter along with cold blood cardioplegia in the adenosine treatment group, but control group patients were subjected to cold blood cardioplegia only. The results were assessed in terms of determining the myocardial arteriovenous difference in oxygen in both test and control group patients. Lactate was also measured before, during, and after aortic occlusion. It was found that there was no significant difference in arteriovenous oxygen and lactate levels between adenosine-treated patients and placebo-group patients. In addition, there was no significant difference in the levels of CK-MB and troponin-T between adenosine-treated and control group patients at different time intervals [191].

Another prospective, single-center, double-blind clinical study was conducted to investigate the effects of intracoronary administration of adenosine on myocardial injury in patients undergoing PCI for correction of STEMI. One hundred and twelve patients suffering from STEMI were involved and randomized into adenosine-treated and placebo-control groups. Adenosine was administered at the dose of 4 mg through the intracoronary route, and effects were assessed in terms of determination of MSI and microvascular obstruction (MVO) by performing cardiac magnetic resonance imaging (MRI). It was found that there was no significant difference in MSI and MVO between adenosine and control group patients. Moreover, TIMI flow grade, TIMI frame count, myocardial blush grade, and ST-segment resolution were similar in both adenosine and placebo group patients after PCI. In addition, infarct size was similar in both groups of patients even after four months [192].

However, in contrast to the above studies, few clinical studies have reported adenosine administration to not show any cardioprotective effects in patients suffering from various cardiovascular disorders [193-196]. A double-blinded study conducted by Garcia-Dorado and coworkers comprised 201 patients with STEMI receiving percutaneous coronary intervention. The patients were randomized into those who received either intracoronary adenosine (4.5 mg) or saline prior to reperfusion. The results of this double-blinded study showed that adenosine failed to reduce the infarct size [197].

Two randomized, double-blinded, placebo-controlled clinical trials had been initiated to study the effect of a partial adenosine A1 receptor agonist, neladenoson bialanate, in patients with heart failure and a preserved ejection fraction (≥ 45%) or heart failure with a reduced ejection fraction (≤ 35%). However, these studies failed to provide significant improvement in heart failure as well as ejection fraction after administration of neladenoson bialanate [198, 199].

Another study was conducted by Quintana and colleagues for testing the hypothesis that adjuvant therapy with a low anti-inflammatory dose of adenosine might prevent reperfusion injury and preserve left ventricular function. For this study, 608 patients with STEMI randomly received infusions of adenosine 10 µg/kg/min or placebo (saline) to be started with thrombolysis and maintained for 6 h. The effect of adenosine administration was assessed in terms of the primary endpoint, *i.e.*, global and regional left ventricular systolic and diastolic function, by using two-dimensional and Doppler echocardiography before hospital discharge. Additionally, the secondary end-point was also assessed in terms of cardiovascular mortality after 12 months of follow-up of hospital discharge. The study failed to exhibit any significant beneficial effect of adenosine with respect to echocardiographic indices of left ventricular systolic or diastolic function [193].

One more double-blinded randomized clinical trial was carried out by Shalaby and colleagues by employing 40 patients undergoing elective coronary artery bypass grafting (CABG). Among these, 20 patients received adenosine (250 μg/kg) in the aortic root after cross-clamping, followed by cold blood cardioplegia. Another 20 patients (control group) received only antegrade cardioplegia. Tissue samples from the left ventricle (from the apex) were taken before and after the CABG. dUTP nick-end labeling (TUNEL) staining was employed for the identification of apoptotic cells. The study concluded both the groups to be identical in demographic data, cross-clamp time, cardiopulmonary bypass time, and weaning time. There was no statistically significant difference in the postoperative cardiac index as well as in hemodynamic parameters after treatment with adenosine [195].

Fokkema and co-workers conducted another study by employing a total of 448 patients suffering from STEMI. 2 bolus injections of intracoronary adenosine (2 x 120 micro g in 20 mL NaCl) were administered to 226 patients and placebo (2 x 20 mL NaCl) to 222 patients. The first bolus injection was given after thrombus aspiration and the second after stunting of the infarct-related artery. The incidence of residual ST-segment deviation, *i.e.* <0.2 mV, between 30 to 60 minutes after PPCI, was considered as the primary end-point, whereas ST-segment elevation resolution, myocardial blush grade, thrombolysis in myocardial infarction flow on the angiogram after PPCI, enzymatic infarct size, and clinical outcome after 30 days were considered as secondary end-points. At the end of the study, there was no significant difference observed in the incidence of residual ST-segment deviation <0.2 mV between patients treated with adenosine and placebo group patients. Moreover, no significant differences were observed in the secondary outcome parameters. Therefore, this study failed to produce any improvement in myocardial perfusion after intracoronary administration of adenosine [196].

## CONCLUSION

Adenosine, over time, has gone through a long journey to become one of the most extensive cardioprotective molecules. Animal studies have very well established adenosine as an important trigger of conditioning techniques. A good number of clinical studies over the last decade have also advocated adenosine signaling as one of the primary events in the cardioprotective mechanism of conditioning techniques. An in-depth analysis of adenosine receptor signaling in cardioprotection will likely provide a better understanding of the role of adenosine in myocardial conditioning. Barring a few studies, most of the clinical trials with adenosine have been found to be successful. Therefore, it may be concluded that the signaling diversity and variable actions of adenosine need to be explored in-depth to translate the successful cardioprotective trials of adenosine in clinical settings.

## LIST OF ABBREVIATIONS

CABG: Coronary Artery Bypass Graft
STEMI: Segment Elevation Myocardial Infarction
PCI: Percutaneous Coronary Intervention
PTCA: Percutaneous Transluminal Coronary Angioplasty
